# Gateway to Recovery: A Comparative Analysis of Stroke Patients' Experiences of Change and Learning in Norway and Denmark

**DOI:** 10.1155/2019/1726964

**Published:** 2019-01-17

**Authors:** Hanne Pallesen, Lena Aadal, Siri Moe, Cathrine Arntzen

**Affiliations:** ^1^Hammel Neurorehabilitation Centre and University Research Clinic, RM, University of Aarhus, Aarhus, Denmark; ^2^Department of Health and Care Sciences, Faculty of Health Sciences, University of Tromsø, The Arctic University of Norway, 9037 Tromsø, Norway; ^3^Centre for Care Research, North, Department of Health and Care Sciences, Faculty of Health Sciences, UiT the Arctic University of Norway, Norway; ^4^Division of Rehabilitation Services, University Hospital of North Norway, 9038 Tromsø, Norway

## Abstract

**Objectives:**

The recovery process is reported by stroke survivors to be a change process fraught with crises and hazard. Interaction with health professionals and others may play a central role in establishing renewed control over life.

**Research Questions:**

(1) How do patients handle and overcome experienced changes after stroke? (2) How do they experience the support to handle these changes during the first year after stroke? (3) How do the similarities and differences transpire in Danish and Norwegian contexts?* Methodology*. A qualitative method was chosen. Six patients from Denmark and five patients from Norway (aged 25-66) were followed up until one year after stroke, by way of individual interviews. The data were analyzed (using NVivo 11) by means of phenomenological analysis.

**Findings:**

The participants described four main issues in the recovery process that impacted the experienced changes: (i) strategies and personal factors that promote motivation, (ii) the involvement of family, social network, and peers, (iii) professionals' support, and (iv) social structures that limit the recovery process. There was a diversity of professional support and some interesting variations in findings about factors that affected recovery and the ability to manage a new life situation between Central Denmark and Northern Norway. Both Norwegian and Danish participants experienced positive changes and progress on the bodily level, as well as in terms of activity and participation. Furthermore, they learned how to overcome limitations, especially in bodily functions and daily activities at home. Unfortunately, progress or support related to psychosocial rehabilitation was almost absent in the Norwegian data.

## 1. Introduction

Stroke is a very common illness. It often leads to lifelong critical disability [1]. All stroke survivors are at risk of complications during recovery, regardless of stroke severity [2]. Patients' initial experiences after the onset of stroke are well described in various studies [3, 4]. The first review of the patient's experiences of stroke was published in 1988 by Doolittle [3, 4]. This was followed in 1997 by Hafsteinsdottir & Grypdonck, in their work “Being a stroke patient” [5], which showed that, in relation to functional abilities, stroke patients often have clear goals for themselves, against which they measure all success and progress in their rehabilitation. Furthermore, stroke patients see recovery as a return to the life they had lived before the stroke, which is different from the view sometimes held by health care providers, which they consider to be “more realistic” [6].

Many studies describe stroke as a sudden interruption of what was otherwise expected to be a normal life course, which disrupts everything related to the activities of daily living [6, 8–10]. In gaining a modicum of control over the body and habitual tasks, they start to be able to cope and shape their training to suit their own requirements [11]. Strategies for motivating and learning are linked in other studies to work identities [12]. Interaction with health professionals and with others in the same situation and the same surroundings plays a central role in enabling them to relearn previously held skills, in finding new ways forward, in achieving a new status, and in recovering self-respect [13, 14]. The recovery process is reported by stroke survivors to be a process of alteration fraught with crises, threats to one's own sense of self, fundamental uncertainty, and the difficult task of establishing a renewed control over one's life and of maintaining a social network and contact with the labour market [15–18]. The suddenness with which the stroke event occurs and the complexities of the recuperation affect patients and family alike [6, 8]. It is, therefore, important to examine how the recovery trajectory and its implications are experienced and to look at the circumstances and the situations of stroke survivors and their families. Both parties often have to reorganize their daily activities.

Processes toward increased involvement of the citizen are described in WHO's definition of rehabilitation [19]: “Rehabilitation is a process aimed at enabling [stroke survivors] to reach and maintain their optimal physical, sensory, intellectual, psychological and social functional levels”. In law, user involvement is rooted in the citizen's right to be involved in her/his own affairs [20, 21].

The phases and transitions of the rehabilitation course have been analyzed [22–24].

A metasynthesis of 18 qualitative studies [25] concluded that individuals' traits, such as perseverance, adaptability, and ability to overcome emotional challenges, can facilitate reintegration into the community, despite persisting effects of stroke. Appropriate support from family, friends, and health professionals seems important [25].

Meaningful activities [26] and innovative solutions to train in real-life situations may help in the process of enabling occupation after a stroke [27] and should be tailored to the individual stroke survivor [25]. The focus should be not only on stroke survivors, but also on their relatives. The existential level of self-management after stroke should also be addressed [28]. These findings are in line with the results in a metaethnographic review [29], in which it was found that the ability of the person to accept their stroke-related problems and adapt their behavior and attitude, by using active decision-making and self-management skills, is key factors in social participation after stroke. Thus, recent qualitative research has identified the importance of understanding community reintegration as a complex process after stroke. This means that stroke survivors need to be supported by family, friends, professionals, and the broader community in order to recreate a valued life, self-identity, sense of belonging, and integration. Furthermore, therapeutic activities should be based on an approach of self-management that is related to meaningful activities and tailor-made to the individual stroke survivor.

However, there are very few studies that have specifically looked at stroke patients' experiences of change and the search for ways to handle and overcome the consequences of stroke, especially in regard to the working-age group of 25-65-year-olds. Several studies have been published in Norway around themes of self and transformation [10, 30–32] and in Denmark relating to self-identity, participating, and learning [22, 33, 34]. Norway and Denmark are in many ways similar in terms of their health systems and rehabilitation trajectories. Rehabilitation after stroke in both settings basically follows the same guidelines, but the organization of rehabilitation programmes is more specialized in Denmark than in Norway [35], and this may affect how stroke survivors experience the recovery process.

The focus of the current paper will be the recovery trajectory experiences of stroke survivors as they unfold in the Danish and Norwegian contexts.

The aim of this study is to explore the following questions from the patients' perspective:

(1) How do patients handle and overcome experienced changes after stroke?

(2) How do they experience the support to handle these changes during the first year after stroke?

And, finally we included an interpretative:

(3) How do the similarities and differences transpire in Danish and Norwegian contexts?

The current study is a substudy of a multicentre study “The NORDA -study”, which is exploring stroke patients' experiences of rehabilitation pathways in Norway and Denmark. The municipal cross-disciplinary rehabilitation offered in Denmark and Norway has already been described [35].

## 2. The Scandinavian Model

The stroke rehabilitation services in Norway and Denmark are founded on the Scandinavian welfare model, which involves free and accessible services to all citizens [36]. During the last 20 years, stroke rehabilitation services in Norway and Denmark have gone through profound organizational and staff developmental changes. In both countries, there are now shorter hospital stays and the municipalities have taken over comprehensive responsibilities for the entire stroke rehabilitation process [37, 38]. Professional case managers with relevant competencies are recommended. Such individualized services may be decisive in relieving relatives, continuing follow-up contact, ensuring psychological support, and linking to other treatment services [37]. A report on brain injury (including stroke) rehabilitation (National Board of Health 2011), based on systematic, critical, and comprehensive health technology assessment, concluded that brain injury rehabilitation in Denmark should be organized across the municipalities, administrative regions, and the state (administrative boundaries) and across professions, such that the rehabilitation services are targeted appropriately, are of uniform high quality, and are coherent for the people involved [37]. In Norway, the Directorate of Health has taken the initiative to elaborate National Guidelines for Rehabilitation after stroke [38]. Likewise, in Denmark, the reform implies that most aspects of the rehabilitation services are now the responsibility of the municipalities [39]. Thus, both countries seem to have legislated for and described a coherent effort that enables high-level rehabilitation services that aim to satisfy the needs of citizens after stroke and support their autonomy.

## 3. Methods

The study adopted a qualitative descriptive design, based on extensive data materials collected from the Region of Northern Norway and the Central Denmark Region. A qualitative approach was chosen to explore the participants' experiences and to identify and search for patterns of ways to handle and overcome these changes during the first year after stroke. The data were analyzed by using a five-step phenomenological analysis, which enables the researcher to enter the world of the participants and gain insight into their thoughts, feelings, and preferences [7, 40].

## 4. Participants

Adult stroke survivors with a clinically confirmed diagnosis of ischaemic or hemorrhagic stroke were included for this study. They had impairments that required further rehabilitation after admission in the one stroke unit in Central Denmark Region or one of four stroke units in the Region of Northern Norway. Participants were selected to represent the stroke group in the working age (25-66 years) and included both men and women. In addition, they were selected so that there was reasonable variation with regard to marital status and participation in rehabilitation programmes. Individuals with communicative impairments preventing them from partaking in conversation were excluded. Six Danish participants were discharged to two communities, while five Norwegian participants were discharged to five different communities. The severity of the stroke incurred by all 11 participants meant that they were offered a public rehabilitation course free of charge. All were hospitalized in the acute phase and most of the participants followed an intensive rehabilitation course at a rehabilitation ward and were subsequently offered a range of services and interventions in the municipality. Two Danish participants went directly from the acute hospital to the municipality service. See Table 1.

## 5. The Interview and Interview Guide

A semistructured interview guide was developed collaboratively by the four authors. The interviews addressed how the participants experienced their level of functioning, significant life changes, social involvement, and goal achievement, as well as their relatives', caregivers', and health professionals' involvement in their recovery process. The interviews were conducted at three and 12 months after stroke. The order and formulation of questions were adapted for each interview situation. The interviewer posed straightforward and easily understandable questions to avoid an abstract linguistic language that might be difficult to comprehend for participants with cognitive difficulties.

### 5.1. Main Themes of the Interview Guide

Three months after stroke:

(i) Concerning getting a stroke and experiencing a change in life. What significance does this have in managing everyday life?

(ii) Concerning learning how to live with the stroke. Identify good learning situations and factors that facilitated the recovery process.

Follow-up interview. Experiences 12 months after discharge from hospital:

(iii) Concerning living a daily life. Looking back on the last year, could you, the health professionals, or others have done something that would have improved how you manage today?

(iv) Concerning aspects of life related to the phenomenon of quality of life and recovery. Has your health condition changed your daily living, quality of life, your plans, hopes, and thoughts about the future?

## 6. Procedures

The interviews [23] were conducted in the stroke survivors' homes. The duration of each interview varied between 60 and 90 minutes. In a few cases, the interviews were supplemented with telephone conversations and email correspondence. In some of the interviews, a family member also took part in a part of the interview. Before the interview got under way, we agreed on a framework and how any next of kin might take part. In most cases, contributions took the form of supplementing the stroke survivor's memories. The interviews were audio-recorded and transcribed verbatim in full.

## 7. Data Analysis

The analysis and interpretation were inspired by Giorgi [7, 41] and carried out in line with phenomenological approaches ([42, 43], using a five-step analytical process. See Table 2.

The comparison analysis was carried out from the beginning of the data collection, with a constant eye on similarities and differences in experiences between the Danish and Norwegian cases. The aim was to systematically conceptualize, categorize, and interpret data from the three-month and 12-month interviews.

## 8. Ethical Considerations

Ethical approval for this study was granted by the Danish Data Protection Agency (Number 1-16-02-66-14) and by the Regional Norwegian Ethical Committee, Health Region South-East (2013/1920) and completed in accordance with the Helsinki Declaration 2008. Participation was voluntary and anonymity was preserved. The names, occupations, and hobbies of the participants have been changed. Oral and written consent to participate were obtained. After the interview was concluded, the interviewee made a final decision as to whether all parts of the interview could be used in the article.

## 9. Findings

For all participants, the sensorimotor impairments that caused compromised walking, reduced hand function, endurance, strength, and coordination problems, were the main focus of the first half of their rehabilitation course. For several of the Norwegian participants, these remained important themes because they continued to challenge recovery. The participants experienced varying levels of fatigue, concentration, and attention problems, often coupled with noise sensitivity, which inhibited social interaction in larger groups of people. Some described that, because they were more sensitive, they could be more easily prone to tears. Communication problems had a negative impact on their social lives. All the participants felt emotionally affected by their illness. Some participants felt depressed at times, while a couple of them described how they believed that, since the rehabilitation, they had achieved a better life than before the stroke. Both the Norwegian and Danish participants experienced positive changes and progress, compared to the early stage of recovery, now that they were more active at home and were participating in family and social events. To some degree, they felt that they could be active citizens and were able to live a meaningful life. However, the changes experienced had also been challenging and had sometimes been overlooked or ignored by the municipal health professionals.

The participants described four issues of importance during the recovery process. The issues had both a positive and negative impact on the changes they experienced and how they had experienced the support to overcome challenges and manage their altered conditions: (i) strategies and personal factors that promote motivation, (ii) the involvement of family, social network, and peers, (iii) professionals' support, and (iv) social structures that limit the recovery process. See Table 3.

### 9.1. Strategies and Personal Factors that Promote Motivation

The informants emphasized goal setting as an important strategy to improve their skills and strengthen the progress of change. Furthermore, they highlighted, using a range of words, concepts, and explanations, that their inherent resources and life experiences had a beneficial impact on how they had managed the consequences of stroke. The analysis showed several subthemes regarding strategies and personal factors to overcome or minimize these limitations and hindrances. Below, the similarities and differences revealed between the Danish and Norwegian contexts will be outlined, consecutively.

#### 9.1.1. Goal Setting: Strengthened the Progress of Change

The informants described the significance they attributed to the therapy and its impact on the progress of change, in both the hospital and municipality. The training and activities thus became contemplative and meaningful, so they could see and feel their progress. A Norwegian (NO) man, number 7, 12 months after stroke (NO 7(12)), expressed* “small goals all the way: … to get my driver's license back … full job … five years on, I can see that my finances are at least getting back to normal … and maybe I can buy the house back.”*

A Danish (DA) man, number 4, 6 months after stroke (DA 4(6)), described the progress of change in another way: “*I also have small victories in my training, because … em … it got better. As well, I'm always climbing by degrees when I do the cognitive training*.”

Both Norwegian and Danish informants talked about how they had set goals for what they would achieve in the short and long term. Several of them also felt that they personally enjoyed being able to feel and measure progress and being able to do so gave them motivation for further training.

The following section will go into a more in-depth presentation of experiences of what they have been doing themselves to overcome and manage new life conditions.

#### 9.1.2. Positive Attitude, Earlier Experiences, Competence, and Mindset

The informants described in different ways how earlier experiences, competences, and their own mindset had a fundamental impact on how to recover after stroke. Several informants described the importance of having a positive attitude, a belief that the situation changes and that by taking action, you yourself can improve your life situation. DA 3(12) spoke about her own attitude in this way: “*I think the most important thing; it's about being positive about things and believing that this is simply going to be ok.”*

Many of the informants were convinced that the various experiences they had come across to overcome challenging phases and tricky life situations had had an impact on how they handled the stroke recovery trajectory. NO 7 described how, earlier in his life, he had been a workaholic and that he tried to escape that with alcohol. As a consequence his son lived for a period of time in an orphanage. The struggle that ensued, to save himself and his son, his believes made him stronger as a person and more competent to cope with the stroke and gave him resources that he was able to draw on. Another man, NO 8(12) months after stroke, expressed the following: “*I was prepared for a stroke, because it's in the family. I have had to manage by myself, alone, since I was 13, because my mother had a stroke then. (…) So I have some experience from that too. I had to grow up at a young age.”*

Everybody defined self-responsibility as an important theme. A woman DA 1(12) said “*You have to take responsibility yourself and hold onto it.”*

Both Norwegian and Danish informants emphasized the importance of having a positive attitude and a belief that they would overcome the challenges. Having experiences from one's earlier life can also be significant and promote resilience. Furthermore, several of the informants also pointed out that one is oneself primarily responsible for getting something positive out of any difficult situation.

#### 9.1.3. Trying Out, Finding New Solutions, Scheduling, and Organizing

All the informants had found new solutions to solving their problems, to varying degrees in different activities and contexts. The analysis of the informants' descriptions showed that they are trying out and experimenting. If they fail, they try to change the situation and try in a new way to find a solution that works. NO 7(12) described “*But, it's more that I've figured out what I can do and what I should try, what might be wise. I may have done it wrong for a while, and then found out a way to make it a little easier.”*

A man, NO 8, had found a way to involve his weak and unused left hand in a number of daily activities. He described how it was a problem for him to use his weak left hand in a natural way while eating, as he involuntarily used his dominant right hand. Therefore, he deliberately used a knife and fork to eat and thus came to use the left hand more. He also gave his left hand the hard work of cutting the food with the knife, all with the intention of developing and improving his left hand function. A woman, DA 5(12), expressed a similar way of activating and involving a weak and less responsive limb, by taking up knitting again, an activity she had previously enjoyed. All informants described the various solutions they had come to, in an effort to overcome their movement difficulties or to involve a body part more in everyday activities, as mentioned above. It was a question of trying, failing, and finding solutions that worked, like a kind of puzzle, or problem-solving process that developed adaptability.

Several of the informants with cognitive challenges had found ways to conquer these. A man, DA 2(6), described how his memory problem appeared and how he had experienced a way to overcome it:* “It can happen that I go to take something out of the cupboard and – what the hell was it I was after. Uff! Then it's gone, you know? So, I have learned to just sit there for a minute and stare into space, and then it comes back to me. Yes, that's strange. The thing about remembering appointments, I prefer to have them written down with times or something like that, otherwise it all goes to pot.”*

All the informants with reduced memory described how they made plans and organized their activities to take account of their memory problems, fatigue, pain, and lack of overview. They used calendars to take notes of appointments and visits and wrote reminders for themselves about what they needed to bring along. In telephone conversations, they noted down any agreements on a writing pad. They wrote down shopping lists before shopping. They divided the day into small sections, so they could save their energy. For example, they might rest before an evening visit, so that tiredness would not overwhelm them or took a painkiller to release a headache after a demanding activity.

#### 9.1.4. Integrating Training into Everyday Life

Having several training sessions, training intensity, and many repetitions of an activity or exercises were some of the factors that the informants described as effective in increasing progress. These factors were often offered as part of the explanation as to why they had been able to integrate training into everyday life situations and settings. Some informants had set themselves a daily routine that included training. For example, a woman DA (1), explained “*I've always done a lot of training and I have been training on a rowing machine and bike every day. There's training in everyday life and lots of exercise, it's the only way forward. Because you cannot do it with the training on its own, the 6 hours you get (name of municipal service), you won't get anywhere.”*

Several informants used their daily routines and activities in a new way, so that the routines themselves became a form of training. NO 11(12) months after stroke said “*Yes, I do the washing up by hand instead of in the dishwasher, just to use the hand and hold things tight. Obviously, all that helps.”*

Others had set themselves a variety of project tasks at home, e.g., carpentry work to make a new wooden floor, repairing small items, or potting around the shed and found that these tasks offered them a way of training.

It seems that the informants were convinced that a large number of training sessions were needed to achieve progress and to overcome the consequences of the stroke. Therefore, they structured their everyday lives to include self-managed training sessions, programmes, or everyday tasks, such as repairs and DIY, or they made changes to their daily chores, so that they were involved in more training than they were offered by the municipality. Some of the informants had even bought special equipment, like elastic training bands, a fitness bike, or a rowing machine to use indoors in case inclement weather prevented them from doing outdoor activities.

#### 9.1.5. Accepting, Avoiding, or Postponing

All the informants experienced issues that were (too) difficult to handle. Some chose to avoid them, while others chose to face them. Others chose to accept the status quo, at least for a while, and maybe do something about it later. The following examples illustrate not so much strategies to overcome the consequences of the stroke, but rather ways to cope and accept that something was too difficult to overcome. It seemed to be a strategy that was used to save energy for other, more essential, activities. DA 4 (12) explained “*It's like I get blockages with regard to what's too difficult (…) because I don't go looking for those situations where I cannot manage.”*

The analysis of the main theme, strategies, and personal factors that promote motivation indicates that it is important to mobilize and display a can-do spirit and believe in one's own ability to find useful solutions through strategies, structure, and organization. It was also found important to accept that some things were too difficult to overcome at the present time and that it would be better not to spend time and energy on these things, but rather to wait and see how the recovery developed.

Both Norwegian and Danish informants presented many different solutions and a wealth of ideas. However, there seemed to be a great deal of difference between Danish and Norwegian data. The Norwegian data described a great many solutions for sensorimotor issues. Conversely, in the Danish data, we found, in addition, many solutions for cognitive deficits and how to cope with existential problems.

### 9.2. The Involvement of Family, Social Network, and Peers

#### 9.2.1. Help from Family Members

Several of the informants described that they got various kinds of help and support from their families. NO 9 said that her son gave her a microwave, so she could make her own meals more easily. She also got practical help from her son by calling him with small requests (e.g., to change a ceiling light bulb or to move heavy objects). Others had, in agreement with their close family members, changed or made adjustments to or reduced the number of daily chores at home (e.g., they bought ready meals).

#### 9.2.2. Significance of Family Involvement

All of the informants talked about the importance of having close family involved in the recovery process. NO 7 explained that he now appreciated his family and felt more humble about the importance of family. NO 7(12) described “*To me it means having a good relationship with my family, a sense of security. That's the most important thing. My family is very close. We stand by each other.” *DA 1 explained how important it had been for her that her husband had not doubted her judgment and had been clear that he would support her in the future. Furthermore, she explained how her family had listened to her stories of anxiety and insecurity in the early phase after stroke: “*Well, you have to have – good support behind you (....) Often, because of the anxiety, it's not easy (coughs) easy to control the situation alone at the start.”*

However, NO 9 was disappointed that her daughter did not show much interest in helping or following up on her situation. The lack of the daughter's presence in the recovery process had made her situation significantly more challenging. The analysis also showed that the roles and responsibilities between, particularly, the spouses had changed. DA 3(12) said* “I get a lot of leeway. I think my wife is fully aware that it is not as it once was. So, I'm actually really glad to have that. Even though I can feel it from her once in a while, she thinks, ahh, now I just have to pull myself up, but she does not knock me over the head with it. It's more like acceptance that things take longer.”*

#### 9.2.3. Conversations about the Stroke and the New Life

All informants had opinions about discussing their illness and problems with other people. Many of the informants discussed their limitations and reduced capacity, both the visible mobility as well as the more invisible factors, such as fatigue, and how these factors had affected their work and leisure activities. DA 3 (6) described “*Well, we talk a lot about it at home. Of course I talk to others about my illness. My colleagues, for example, know what has happened and also ask about it. And I'm very open about it (...) Anyone who asks about it, they definitely get an answer. There is nothing at all that's difficult to talk about or anything embarrassing, nothing like that.”*

However, NO 7, 8, and 10 highlighted that they did not discuss the illness or its consequences with their families. NO 7 said “*And I have greatly appreciated that (referring to the absence of conversations about his stroke). They do not stand there trying to be kind and help. They can see every time I struggle, but they sit there until I ask for help*.* Then they help*. And, NO 10 simply said:* I speak as little as possible about illness*.” Furthermore, they did not want to burden others with their problems or bring up concerns in conversations, when they visited relatives or had a family get-together.

As illustrated above, the material presents three different opinions about whether and how other people should be involved in discussions about the participants' illness and how they managed their poststroke life. Some wanted to talk as little as possible about the illness and the poststroke situation. Other stroke informants wanted to set some limitations on their relationships and only joined a conversation if they were invited to do so. Finally, there was a group of informants who found it important to be as open as possible and to discuss their situation with everyone who showed an interest. The findings also revealed that the Danish informants were more likely to open up and discuss their new life situation than were the Norwegian informants.

#### 9.2.4. Comparison and Interplay with Peers

Finding solidarity among peers in the same or similar situation seemed to bring status, acceptance, and new insight into one's situation. All of these things seemed to be treasured by the stroke informants: NO 7(12) described “(…)* and no matter how bad it is for you, well, it's no comfort, but there are those who have it worse. It's not that it's a comfort, but it does something, things are easier to swallow and accept (...). There's not really much reason to feel sorry for yourself.” *Being able to measure one's own situation against that of others and acknowledge that, on some points, others were suffering more than oneself could help towards a resolution and reduce self-pity.

DA 1(12) said “*I mean, it is still very important, because just the feeling that you do not feel different, even though the leg swings a bit and drags a bit and you stumble over your words sometimes. That means a lot!”*

Being in interaction with peers also seemed to increase the informants' acceptance of normality and diversity, so that having visible impairments seemed to have less impact on their self-esteem. DA 4(12) related “*That you can sit and feel quite high on other people's successes. That gives you something. Also it gives you motivation for yourself. Well, he can do it, so I can bloody well do it too, so (...) not because you have to compete, that you do not go over the top, but you have to think to yourself: Can I just do a little more than I could yesterday? I mean, push yourself a little further, a little bit, but no more than you can.”*

Formal interaction, such as group training or other informal social activities with peers, gave the informants a kind of freedom to develop friendships, a sense of belonging, and self-perception and inspired them to pursue their own initiatives. DA 2(3) explained his experiences of participating in a stroke training group in the municipality: “*Yes, that's where there's a free space, with 7 or 8 people (...) And they're almost like me, yes, more or less (...) they have had a stroke, and you cannot see (...) much physically different about them, so yes, that's a good place.”*

These interactions could also facilitate good conversations about issues that could be intimate or delicate to varying degrees. DA 4(3) said “*Yes, we got on very well socially (re: fellow patients at the hospital). We had a really good group, and I'm still in contact with them. In fact, what we did in my group was, that we called for a meeting about whether to do something more collectively. And that's because we could play games, we could just sit and talk, we could watch television together, we could do whatever (…) It provided the opportunity to have some good chats about: “It's not going so well”, and about lots of things.”*

For most informants, the fact that they could measure themselves (progress, relapse) and their own progress motivated them to continue training and gave them a sense of hope and progress. They assessed who they were as people, and their status, in relation to the time before the stroke, to their age group and to everyday tasks. Drawing comparisons with their stroke peers gave them courage, enthusiasm, and a kind of can-do spirit. Unfortunately, some Norwegian informants also described how they compared their lives before stroke with the current situation and dwelled on the negative consequences of the situation. Furthermore, the Norwegian informants did not seem to have been offered any formal or informal setting where they could interact with peers during the course of rehabilitation. In particular, these social interactions were absent in municipal rehabilitation.

### 9.3. Professional Support

The data material revealed that professional support was essential to the recovery process and several subthemes arose from the data.

#### 9.3.1. Cooperation with Health Professionals

In the Norwegian material, the main focus of cooperation seemed to centre round the physical training. The informants described that they had received the treatment they needed, especially physiotherapy and that it had been specific and tailored, rather than off-the-peg, and chosen and carried out with care.

NO 7(12), in responding about hospital rehabilitation services, related: “*Well, I'm thinking especially about physiotherapy. It was very specific. (...) so there was nothing held back. *Overall, the informants experienced that they were pushed enough. The therapists were aware of the patients' limits and taught them to respect their own limits. NO 7 continued:* They pushed me a little. And I think that's very good. But at the same time, I'm talking specifically about the physiotherapist, so he pushed me, but at the same time, it was over my limit. I cannot do that anymore, you can do it and not just put an end to it, but try it out. Very early on, I had the feeling that he knew what he was doing, if I can put it like that.”*

Both Norwegian and Danish patients were given home assignments, especially when they received municipal rehabilitation. Most of informants were pleased with this service; however, a few Norwegian informants experienced the amount of training to be excessive, while the commuting also felt exhausting, as described by NO 9(12)*: “The way I see it, I put so much effort into the daily tasks at home, that I have enough training in that. So, therefore, I feel it's unnecessary to go to a physiotherapist once a week.”*

The informants described the cooperation with health professionals as unproblematic and they recognized that the staff had great professional insight and understood how to apply the training. Unfortunately, there were also some cases in the Norwegian data where the volume of training was not adapted to the patients' situations.

#### 9.3.2. Individual Customization

The health services seemed to be individualized to some degree, and the patients had an influence over various aspects of the interventions. NO 8(12) stated “*In the municipality, I can get five sessions a week. I chose them to come home to my place four times a week, and then I would be on my own (training alone at home) one day a week.”*

The informants described that they had quite a high degree of influence and that, over time, they themselves gained some control over their own activities. NO 10(12) related:* “Maybe I've got a little more handle on it now. But now there are only two (therapists). That's how it is. The speech therapist and the physiotherapist. It's just those I have contact with. And, really, I manage my time myself. It's going great.”*

The Danish informants expressed greater enthusiasm than the Norwegian informants, especially with regard to the professionals' ability to individualize, “read”, and understand them. DA 6(9) experienced the physiotherapist as follows: “*She could also read me quickly. And what she could push me to do. If she could push it a bit further, she did it. So, she was amazing.”*

Another Danish informant, 4, described that she and the therapist together found useful solutions, talked and reflected on them. Furthermore, the occupational therapist supported her to test these in other contexts, as well: “*She has been amazing. Absolutely fantastic, too, because she is involved in a lot of different areas…. So it's a good structure she has taught me. And then we have, so I really have, together with her, found out so many practical things about, which have been good, em, in relation to children and how to say things and things like that.”*

DA 1 (3) described a very close relationship with the therapist who, during the scheduled sessions, was very good at feeling her mood, although she stated that sometimes the sessions were more about talking than training: “*Both the Physio and the OT were extremely good at challenging me, and at times I thought it's not necessary, I can figure it out, but it's good to get it little ‘just try to do it'.”*

#### 9.3.3. Support or Absence of Support from Health Professionals, Especially in Municipal Rehabilitation

Overall, the Norwegian informants gave many descriptions of incomplete rehabilitation courses after returning from the hospital. There was the waiting time where they themselves had to take action to initiate an intervention. It seemed that few staff groups (they comprised primarily physiotherapists) were involved in the process, which meant that rehabilitation services had a narrow focus. There were also mistakes in documents made on the basis of patient records. Very few Norwegian informants had supplemented their municipal rehabilitation with an intensive rehabilitation stay far away from their homes. NO 11(12) was satisfied with his course, even though he also experienced how the therapists did not support the way he needed it:* “But in spite of everything it was the best. That's what I appreciated there. Also the trips we took at the weekends. Then we got to shop a bit. And we went down for a quick visit, to the cafe there. Then we did a sightseeing tour to the border with Russia. I really appreciated it, and also, at the weekends we got some extra good food (laughs).” *

The Danish informants were involved in a range of group interventions that supported them in ways beyond the training of their physical skills. The professionals (both in hospital and municipal rehabilitation) arranged, e.g., ad hoc group activities and organized and framed the activities so that patients were given a range of tasks to support them in developing the skills (cognitive as well as social) that had been affected because of the stroke. DA 6 (3) related: “*They (the health professionals at the hospital) were also constantly focused on you not just sitting in your living room, you know? They created that patient group, and encouraged you to join. It wasn't that you had to, but they think that, together with a … oh, we had one person with us who wasn't a patient. Like a kind of chairperson. Then we were to sit and tell each other about ourselves. I think it was amazing.”*

Descriptions of progress or support related to psychosocial rehabilitation were vague in the Norwegian data. Those descriptions were not directly listed as topics focused on rehabilitation interventions or approaches and seemed particularly lacking in the municipal services. None of the health professionals seemed to assume responsibility for or took action to deal with these issues in the Norwegian courses.

#### 9.3.4. Involving the Relatives

The Danish relatives received support throughout both the hospital and municipal rehabilitation. They were invited to take part in conversations, status meetings, had phone calls, and were invited to relatives' meetings through the user organization Hjernesagen.

DA 3(3) described how significant it had been for him that the health professionals had perceived him and his wife as a unit and that both needed support. It had given them confidence and clarity that the wife was so well informed about the consequences of his stroke. His wife said* “Well, I'm out at (name of hospital). I talked a lot with (name of health professional) who also called me up to hear how I was, how it was going, and how I was managing, and there was a lot of openness and understanding and care, and all of that, I really liked it and in the municipality, they offered me something. I was just to get in touch if there was anything, you know? But then I got the other (psychology sessions) from my workplace and then, oh, I said yes to that, because they (staff from her workplace) had asked a few times.”*

The Norwegian patients did not receive similar support in their rehabilitation process. They had to conduct outreach themselves in the hope of finding support in their own social networks.

### 9.4. Social Structures That Limited the Recovery Process

In both the Danish and Norwegian cases, several limiting factors were reported that had a negative effect on the recovery.

#### 9.4.1. Return to Work: a Particular Issue

The informants in this study were all under the age of 65. In Scandinavian culture, this means that they are still considered to be of working age and staying connected to the labour market is of particular importance. The Norwegian cases were supported by NAV, Norwegian Labour and Welfare Administration, a publicly owned employment and inclusion company. The informants had varying experiences of their support; most had good experiences of the customized work, while NO 7 felt relieved when the course was completed and stated that it was an agency that they should get rid of as soon as possible. Twelve months after stroke, DA 2 was granted early retirement and was pleased with that. DA 3 began after about six months after stroke to return gradually to his old job (beginning with two shifts of two hours per week), and six months later he had returned to his full time job. He described it as follows: “*And I have the same areas of responsibilities and… oh, we had a staff meeting on Monday and they asked if I would take the minutes, because I'm used to it, being the regular minute-taker. And in fact, it was really nice to have to keep up with writing the minutes and take part in… but I had thought, like, a bit, that it was sort of a … could easily be a test. But it actually went really well.”*

DA 4(12) had a more sceptical and socially critical perspective on getting back into the labour market: “*No. I mean, I have a disability now, as they say, as she, the neurologist at (name of service) says: “Your future career will be hard. It will be really hard”, she says. “But you can easily work. The (labour market) is not geared up for people who have an injury”, she said. “Not at all! It just isn't.” It is good to know that in advance! So, where you think, and she says that also to be realistic, and I know that, and it's not because I'm able to hear the truth, but it's bloody hard, you know*.”

This skeptical attitude of the neuropsychologist was presumably intended to prepare the patient for the challenging task of returning to the labour market and encourage her not to give up finding a job that would suit her.

In both countries, participants had the opportunity to get professional support and were offered a range of job training courses before returning to full-time or reduced-time employment. If they were no longer deemed capable of working, early retirement could be considered. Although some of the participants had undergone a seamless course regarding work rehabilitation, as in DA case 3, where work rehabilitation was implemented in the municipal rehabilitation, both the Danish and Norwegian data revealed that several of the informants felt this part of the rehabilitation was insufficient and played against instead of with the patient.

#### 9.4.2. Getting Out and About: Transport and Driving Ban Limited Recovery

Being able to walk about out of the house, visit friends and family, or go shopping whenever it suited them had significantly changed after the stroke and had disturbed their recovery. All informants described in different ways how their affected mobility and transportation and the driving ban had had a major influence on their progress after hospitalization and their efforts to re-establish a normal everyday life after stroke.

NO 9 (12) related: “*I have loved going out and seeing people and meeting people. And even in the daytime I do not get to do that, because – before – I went to town almost every day by bus for a cup of coffee and to enjoy myself. But no, now it might not be even once a week that I get to town, and then it's just to do the shopping, and it is not so enjoyable*.”

The informants described that they did not often go out to social events or talk with other people and that had reduced the quality of their lives. They spent more time at home, watching television, or lying on the couch, which made life more boring. All the informants had had a driving licence before the stroke. After the stroke, they all had a period of 6-12 months' driving ban. All stroke patients in Denmark and Norway, by law, have at least 3 months refrain from driving. Everyone was adversely affected by this long period of ban, but it was particularly inconvenient for those who lived in the countryside and/or lived alone. DA 1(6) said “*A driving ban is of great significance – the experience of not being able to get out when you want, that will be difficult to handle, especially when, like me, you live in the countryside.” *Furthermore, several of the informants described how challenging it was to ask others to drive them and thus bother family, neighbours, and friends and make them have to change their plans. Uncertainty about the driving ban had a lasting emotional effect on some of the informants. For DA 5, who had worked as a taxi driver, repurchasing her business licence had altered her mood and reduced her courage.

The health system had, to a certain extent, taken account of the ban and arranged for transport to the municipal training. However, participation in family, cultural or other events was significantly reduced during the time they were banned from driving. For most of the stroke survivors and their close relatives, the ban had been temporary. Looking back, they viewed it as an unpleasant experience that had delayed recovery.

The analysis revealed that the Norwegian, and to some degree also the Danish, informants were prevented from participating in social events and being active in society, thus making them more house-bound.

## 10. Discussion

The aim of this study was to explore the following questions about recovery, from the patients' perspective. (1) How do patients handle and overcome experienced changes after stroke? (2) How do they experience the support to handle these changes during the first year after stroke? And, finally, we included an interpretative question (3) how do the similarities and differences transpire in Danish and Norwegian contexts?

### 10.1. Gateway to Recovery

The participants described how both minor and more complex changes had a significant impact on and presented great challenges to their everyday lives. Although they would continue to have to deal with impairments that had changed their life situation forever, they also experienced that they had learned to manage and overcome most of the challenges regarding being active and participating in social events. Problems in getting out and about, transport, and the driving ban had limited recovery profoundly, but, looking back one year after stroke, these restrictions were labelled as temporary. They had delayed recovery and for a temporary period had narrowed the gateway to recovery. Although the recovery process and the contexts were different, four themes about learning emerged as significant across the two Scandinavian countries: the first three themes are strategies and personal factors promoting motivation; involvement of family, social network, and peers; and professionals' support. Considered together, they seemed to have a positive influence on opening up the gateway to recovery. The fourth theme, social structures that limit the recovery process, which relates to the subtheme* return to work,* revealed that several of the Danish and Norwegian informants felt that they did not get the relevant support that could had helped them to return to some kind of work. So this part of the rehabilitation course was insufficient and narrowed the gateway. Clarification of stroke patients' work ability, structured guidance, and slow start-up at work, as we saw it in example with the Danish patient number 3, seems conducive to recovery.

However, the variances in the municipality services and interventions revealed some conspicuous differences between the Danish and Norwegian contexts. The main differences seemed to be the absence in the Norwegian context of professional support in the municipal services and a lack of interventions to address stroke survivors' psychosocial problems or to support the family, together with an absence of environmental factors. These might include formal or informal settings where survivors could interact with peers during the rehabilitation course, especially in the municipal services, that could facilitate learning and help them manage their changed life situation.

### 10.2. Rehabilitation versus Recovery

Results from this study show that traditional physical rehabilitation and, to some extent, the cognitive rehabilitation are satisfactorily viewed from the patients' perspective. They feel involved in rehabilitation goal setting and can gradually take over and carry out their own everyday workouts, employing inventive and creative solutions.

Psychological rehabilitation seems to be present in the Danish context, while it does not seem to be present in Norwegian context, particularly in the municipal rehabilitation.

Important, but often overlooked, aspects of* psychosocial *motivational work are absent in Norwegian data, e.g., personal factors, such as emotional and cognitive resources, coping, self-efficacy, or identity, that could had been used in social settings to achieve a satisfactory and meaningful quality of life. Similar results were found in Green et al. and Walsh et al.'s studies [6, 25]. Health care providers view recovery in a way that is measurable, in terms of isolated and discrete turning points. Whereas, in the eyes of the patients, recovery means a return to previously valued activities [5]. These findings are in line with current descriptions in the subthemes* trying out, finding new solutions, scheduling and organizing*and* integrating training into everyday life*. Several studies [8, 44, 45] have found that the trajectory of stroke recovery may necessitate a reevaluation of life plans, a rethinking of priorities, and an integration of resulting disabilities into current and emerging life situations, for both stroke survivors and their caregivers. Similar adaptations emerged in the three subthemes:* accepting, avoiding, or postponing; significance of family involvement; *and* conversations about the stroke and the new life. *In many of the cases, these adaptations were compounded by transitions from the hospital setting to home and everyday life. Adaptation is a core theme in a metasynthesis [8] from 2008, which includes nine studies from the period 1993-2004. Salter et al. concluded that the sudden, overwhelming transformation of stroke creates the background for loss, uncertainty, and social isolation. However, stroke survivors may move forward through adaptation towards recovery [8, 45]. The patients in the current study described how strategies and personal factors had a fruitful impact on progression, but also described the importance of support from peers to find new ways of doing, thinking, and coping. That kind of support was also head lighted in a Canadian study [45], which facilitates the rethinking of priorities, the integration of remaining disabilities into a new life situation, and the recognition of the recovery as a process of transformation seemed to be lacking in the cases described by the Norwegian informants in the present study. Similar results were found in a Danish study exploring stroke survivors' experiences of living five years after stroke; there was a greater focus on the aspects of change and the redefinition process [46]. The study recommended that the aspects of changes should be taken seriously in the form of a higher focus on change-oriented approaches in rehabilitation efforts, in terms of disability, identity, and everyday life [46]. These recommendations seemed to have been followed in Denmark and implemented in the rehabilitation practice, in both hospitals and municipalities. Such recommendations have been addressed in several other studies, as described in the introduction to this article [26, 29, 44, 45].

It seems obvious that stroke rehabilitation, its approach and interaction with the patients and relatives, should be based on recovery-based thinking and concepts. Perkins (2012) [47] defines recovery thus:

Recovery is not something that services or professionals do; it is a personal journey of discovery: making sense of, and finding meaning in, what has happened; discovering your own resources, resourcefulness and possibilities; building a new sense of self, meaning and purpose in life; growing within and beyond what has happened to you; and pursuing your dreams and ambitions.

All the stroke individuals participating in this study were involved in a process of redefining themselves regarding their reintegration into the community, and some of them were supported by the professionals to undergo this transition.

### 10.3. Recovery–Discovery

A Danish psychiatrist [48] recommended using the term “discovery” to describe the discovery journey following an acquired illness, rather than the word recovery. A discovery journey contains unmatched opportunities for development. It is about “discovering” what emerges and understanding contexts. It seems that, more than the Norwegian rehabilitation professionals, their Danish counterparts used such a “discovery” perspective. The Danish rehabilitation professionals seem to have invited and supported patients and relatives to discover and to develop their own ways. Their perspective was not exclusively a medical and physiological perspective focusing on predictions and treatment of impairments. However, a Norwegian study [49] of lifestyle groups after stroke concluded that the participants were active contributors in the groups and pushed each other and themselves regarding involvement in meaningful occupations. This active participation seemed to bring the participants' resources into focus and contrasted with the frequent negative perceptions of people after stroke as “victims” [49]. However, this philosophy does not seem to be practised in the Norwegian cases. The change-oriented approach in rehabilitation services, also called transformative learning, is about how to understand and develop your prerequisites to relate to yourself, your existence, and your surroundings, learning through change and transformation. You change the meaning or understanding of something you have previously acquired, possibly at the same time adding something more. A former study [44] concluded that transformative learning can offer insight into how stroke survivors learn, rebuild competence, and re-engage in valued activities. Rehabilitation practice that addresses and supports autonomy, social connections, risk taking, adaptation, and hope among stroke survivors may help individuals re-establish personally valued activities after stroke [45].

**Figure 1:**
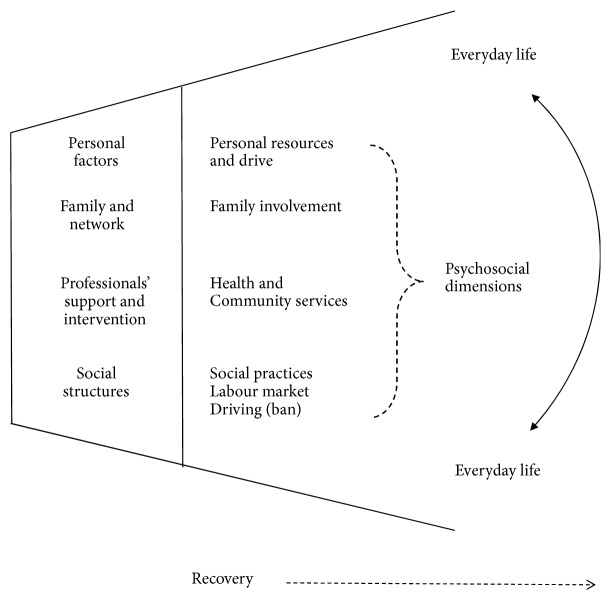
Gateway to recovery.

This comparative study reinforces previous studies that highlighted the importance of an approach that facilitates individuals' perseverance, adaptability, and ability to overcome emotional challenges and help reintegration into the community, despite persisting consequences of stroke. Appropriate support from family, friends, and health professionals seems to be factors interacting with each other in initiating and facilitating the transformation process and thereby widening the gate of recovery. See Figure 1.

According to Illeris [50], the change can best be related to changes in the learner's identity: the understanding of who you are, cognitively, emotionally, socially and as a participant in society. The current study shows that practising such a learning approach can widen the gateway to recovery. That kind of learning approach, employed with a view to serving patients' recovery and transformation, is an area that requires systematic effort, if it is to be implemented in the field of rehabilitation.

Research that develops and examines the efficacy of such learning approach and programmes is recommended. The next step in a Norwegian-Danish collaboration could be to develop and evaluate a return to work program based on the above-mentioned principles.

*Limitations and Strengths. *The concepts of transferability, credibility, dependability, and confirmability were used to evaluate the scientific trustworthiness of the study [51–53]. To increase the credibility of the study, triangulation methods [54]) were utilized. However, the limited sampling of the current study and excluding patients with limited speech ability reduced the transferability to all stroke patients.

The interview guide was prepared jointly by two Norwegian and two Danish researchers and was used as a checklist in all interviews. However, it is important to mention one critical methodological consideration that may affect the results of this study. Generally, there is a lack of in-depth questions in the Norwegian interviews that would reveal the importance of the change and learning perspective, such as these questions “what does the informant mean by expressing that he cannot use his arm as he could before?” “What has or could have facilitated progress?” The Danish interviewers seem to have been more proactive in exploring quality, dimensions, and aspects that influence learning and change and follow up on unanswered aspects of an important upcoming theme.

## 11. Conclusions

Both the Norwegian and Danish participants experienced positive changes and progress on the bodily level, as well as in activity and participation. Furthermore, they learned how to overcome limitations, especially with regard to bodily functions and daily activities at home. Unfortunately, progress or support related to psychosocial rehabilitation was almost absent in the Norwegian data.

## Figures and Tables

**Table 1:** Participants' characteristics.

Case	Sex	Age	Country	Marital status	Work after stroke	Citizens in municipality
1	Fe	<60	DA	Cohabiting	Retired	61,000

2	Ma	<65	DA	Cohabiting	Retired	61,000

3	Ma	<50	DA	Cohabiting	Full-time	61,000

4	Fe	<40	DA	Cohabiting	Work-training	48,000

5	Fe	<55	DA	Cohabiting	Work-training	48,000

6	Ma	<55	DA	Cohabiting	Work-training	48,000

7	Ma	<50	NO	Single	Work-training	4,800

8	Ma	<65	NO	Single	Retired	72,000

9	Fe	<50	NO	Single	Retired	9,500

10	Ma	<45	NO	Cohabiting	Work-training	3,500

11	Ma	<60	NO	Cohabiting	Retired	5,500

**Table 2:** Five steps in the analytical process inspired by Giorgi [7].

1	Reading and rereading the whole interview in order to gain a sense of the whole	A research assistant undertook the transcription of each interview. Each interview transcript was then read by the first author, after which the interview was played again, to ensure that the transcription was accurate.

2	Natural “meaning units,” as they were expressed by the interviewees, were identified by the researcher	The data were transferred into NVivo 11. The data were analyzed in depth, using a phenomenological method to trace thematic patterns of how to handle life changes.

3	The dominating themes in the meaning units were identified. The researcher tried to thematize from the interviewed person's point of view, as the researcher understood it	Meaning units were organized and gradually transformed into categories. Firstly, the data were separated into Danish and Norwegian sets and, secondly, similarities and differences were noted.

4	The meaning units were questioned based on the research questions	The data were described in a final set of themes and subthemes that responded the research questions

5	Condensing the nonredundant themes into descriptive statements	The final results were cogenerated by the final author and discussed with coauthors, after which it was considered to constitute essential knowledge

**Table 3:** Ways to overcome and manage altered conditions.

Main theme	Subthemes
**Strategies and personal factors that promote motivation**	*Goal setting – strengthening the recovery process *
	*Positive attitude, earlier experiences, competence and mindset *
	*Trying out… finding solutions … scheduling and organizing *
	*Integrating training into everyday life *
	*Accepting, avoiding and postponing?*

**The involvement of family, social network and peers**	*Help from family members *
	*Significance of family support *
	*Conversations about the stroke and the new life *
	*Comparing with others and interplay with other peers*

**Professionals' support**	*Cooperation with health professionals *
	*Individual customization *
	*Support or absence of support from health professionals *
	*Supporting the relatives*

**Social structures that limited the recovery process**	*Return to work – a particular issue in the recovery process *
	*Getting out and about – transport and driving ban limiting recovery*

## Data Availability

A protocol is available in Danish which sketches the research process and contains all the transcriptions. It shows the full transcription of the empirical data material, including the quotations presented in the results of the study.

## Additional Points

*Clinical Implications*. Psychosocial dimensions and transformative learning approaches to rehabilitation should be addressed in the clinical setting to achieve successfully recovery.
